# The Trendelenburg Position and Cognitive Decline: A Case-Control Interventional Study Involving Healthy Volunteers

**DOI:** 10.2196/11219

**Published:** 2019-01-15

**Authors:** Parveen Vitish-Sharma, Charles Maxwell-Armstrong, Boliang Guo, Crystal Yick, Austin G Acheson

**Affiliations:** 1 Nottingham University Hospitals NHS Trust Nottingham United Kingdom

**Keywords:** POCD, Trendelenburg, cognitive function, laparoscopic

## Abstract

**Background:**

Postoperative cognitive decline (POCD) is defined as a new cognitive impairment arising after a surgical intervention. Aspects of cognitive function can be assessed using various validated cognitive function tests including the N-back task, the Stroop task, and the lexical decision-making task (LDT). There is some concern that prolonged Trendelenburg positioning during laparoscopic colorectal surgery may cause POCD.

**Objective:**

The objective of this study was to assess the effect of time spent in the Trendelenburg position on cognitive function.

**Methods:**

Volunteers were placed in the Trendelenburg position for 3 hours and, then, supine for 30 minutes. Validated cognitive function tests including 1-, 2-, and 3-back tasks, Stroop test, and LDT were performed at baseline and every 30 minutes after Trendelenburg positioning. Cognitive decline was defined per the International Study of Postoperative Cognitive Dysfunction trial: a decrease in accuracy from the volunteers’ baseline or an increase in response time from the volunteers’ baseline by >2 control group SDs.

**Results:**

We recruited 15 healthy volunteers (8 males, 7 females) with an average age of 69 years (range 57-81) and average body mass index of 27.7 kg/m^2^ (range 20.9-33). Accuracy remained within 2 SDs at all time points. An increase in response time did occur, and of 15 participants, 3 (20%) showed cognitive decline in the Trendelenburg position after 30 minutes, 4 (27%) after 1 hour, 5 (33%) after 90 minutes, 4 (27%) after 120 and 150 minutes, and 6 (40%) after 180 minutes. On moving to a supine position, 33% (5/15) participants showed cognitive decline.

**Conclusions:**

The results of this study indicate that Trendelenburg positioning leads to cognitive decline. This may have implications for patients undergoing prolonged Trendelenburg positioning during laparoscopic colorectal surgery.

## Introduction

Impairment of cognitive function following surgery has been recognized since the 1950s. Postoperative cognitive decline (POCD) is defined as a new cognitive impairment arising after a surgical intervention [1]. It is a subtle disorder of thought processes, which may influence isolated domains of cognition such as verbal memory, visual memory, language comprehension, visuospatial abstraction, attention, or concentration [2]. POCD can lead to increased hospital stay, higher readmission rates, impairment of daily functioning, delayed return to work or normal level of functioning, and dependency on government economic assistance post discharge from hospital [3]. It can affect patients at any age but was shown to have a longer and more significant effect on daily life activities and return to work in patients aged >60 years.

Trendelenburg positioning is commonly used during laparoscopic colorectal surgery to allow the use of gravity to move the small bowel out of the pelvis and provide the surgeon with adequate view. The degree of tilt and time spent in these positions vary depending on the type of resection and complexity of the case. The gravitational effect of the Trendelenburg position is thought to divert blood away from lower extremities and increase central blood volume [4]. This increases cerebral blood flow and intracranial pressure by impairing venous outflow from the brain, increasing hydrostatic pressure within the cerebral vasculature and pushing fluid into the extracellular spaces. After being in a steep Trendelenburg position for a prolonged period, significant cerebral perivascular edema can develop. All of these can cause impaired cerebral perfusion and cerebral edema [5]. If significant cerebral perivascular edema develops, the effective cerebral perfusion may get significantly reduced [6], resulting in impaired tissue oxygenation and leading to cognitive decline [7]. This effect is likely to be exacerbated if a pneumoperitoneum is used, which would further increase central venous pressure.

The N-back task is used as a measure of working memory and executive function [8]. It is favored because the ability to change the value of “N” gives researchers a reliable way of altering the processing load of the task [9]. The Stroop test provides a paradigm case of attention and inhibition. The Stroop effect is recognized as the inclination to say the word presented rather than the color that it appears in. A delay in response is usually seen when the color of the word does not match the meaning of the word (an “incongruent stimulus”) [10-12]. Lexical decision-making task (LDT) primarily tests the language aspect of cognitive function. Executive functions (decision making) are also tested in this task because a quick decision must be made as to whether the letters form a word or not [13]. The aim of this study was to assess the effect of the amount of time spent in the Trendelenburg position on cognitive function.

## Methods

### Ethics Approval

This case-controlled interventional study involving healthy volunteers was reviewed and approved by the University of Nottingham Research Ethics Committee (reference #: N14082014 SoM GI Surgery NDDC).

### Recruitment

We recruited healthy volunteers aged >18 years. Volunteers with pre-existing cognitive impairment (either a pre-existing diagnosis of dementia or poor performance in baseline tests), smokers, those unable to read or understand English, those with visual impairment, or those who refused to give written informed consent were excluded from our study.

After practicing each test 3 times (to allow for the learning effect of repeated tests), a baseline performance for each cognitive function task was recorded while sitting for 1-back, 2-back, and 3-back task; Stroop test; and LDT.

**Figure 1:**
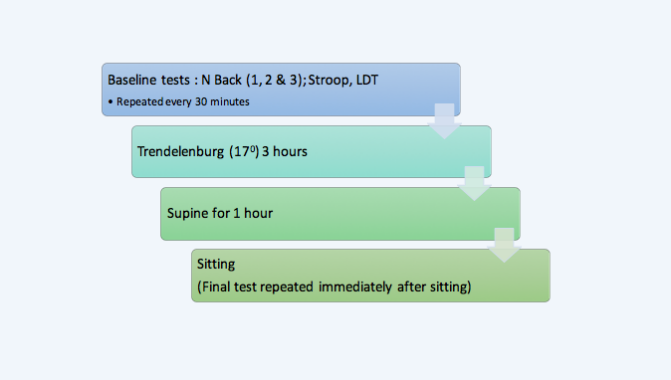
Flow diagram of position changes during the study. LDT: lexical decision-making task.

The volunteers were then placed in the Trendelenburg position at 17° head-down for 3 hours, moved to supine position for 1 hour, and then asked to sit up. The above tests were then repeated after 30 minutes, 1 hour, 1.5 hours, 2 hours, 2.5 hours, 3 hours (volunteers were moved to supine position after 3 hours, so these tests were performed immediately after volunteers moved to supine position), 3.5 hours, and 4 hours (after 4 hours, the volunteers were asked to sit up in a chair, and the tests were performed immediately after they sat up; Figure 1). Between 90 minutes and 2 hours, 5 of the volunteers took a “toilet break.” The duration of toilet breaks ranged between 2.5 minutes and 4.8 minutes including walking to and from the toilet. This break was unavoidable because our volunteers were awake.

### Control Data

To allow for the continued learning effect that may mask the cognitive decline, a control group was recruited to provide control data for analysis. Accordingly, 5 volunteers repeated each test 4 times. Each test was analyzed individually, and the mean and SD values were calculated for a change in both accuracy and response time (RT) between the third and fourth attempt of the tests. The mean change was taken to represent the learning effect for the tests, which is known to occur when the same tasks are repeated multiple times.

### Statistical Analysis

#### Power

A statistical power analysis was performed for sample size estimation based on data from Brookes et al who compared the effect of increased task difficulty on magnetoencephalography activity. They detected a mean accuracy of 98% (SD 2%) for the 1-back task and 91% (SD 8%) for the 2-back test. With an alpha of .05 and power of 0.80, the projected sample size needed with this effect size (STATA 14.0) was n=10 [14]. We, therefore, chose to recruit 15 volunteers to our study.

A repeated measures analysis was performed to compare the change in accuracy and RT at each time point. For each volunteer, the accuracy and RT at each time point was subtracted from their own baseline results. A repeated measures test was then performed using STATA.

To assess the percentage of volunteers who suffered cognitive decline from their own baseline performance, the International Study of Postoperative Cognitive Dysfunction (ISPOCD) definition was used [15]. The baseline for the volunteers was subtracted from their own results at each time point; furthermore, the learning effect was also subtracted from this change in “test score”. This result was then divided by the SD of the control group to give a Z score. A large positive Z score (z>1.96) showed a deterioration in cognitive function from baseline for accuracy, and a large negative Z score (z<−1.96) showed the same for RT [16]. This, therefore, allowed each time point to be compared directly to the volunteer’s own baseline performance. Therefore, any decline in performance indicated a decline from the volunteer’s own baseline performance.

## Results

A total of 15 healthy volunteers (8 males and 7 females) completed the study. The average age of the participants was 69 (SD 6.98) years and average body mass index was 27.7 (SD 3.4) kg/m^2^. Tables 1 and 2 show the results of repeated measures analysis of the change in accuracy and RT from the volunteers’ own baseline, respectively. The percentage of volunteers with cognitive decline from their baseline was analyzed (Table 3).

**Figure 2:**
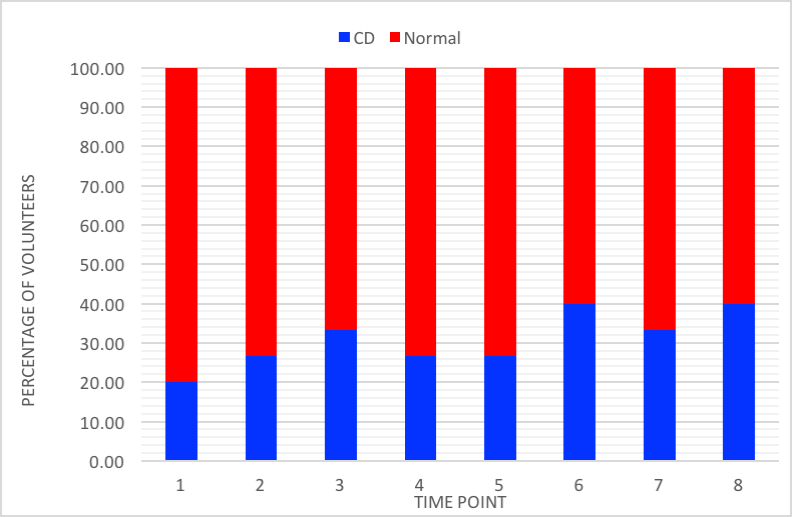
Percentage of volunteers with cognitive decline at each time point. 1: Trendelenburg 30 minutes; 2: Trendelenburg 60 minutes; 3: Trendelenburg 90 minutes; 4: Trendelenburg 120 minutes; 5: Trendelenburg 150 minutes; 6: Trendelenburg 180 minutes or supine 0 minutes; 7: Supine 30 minutes; 8: Supine 60 minutes or volunteer sat up. CD: cognitive decline.

Figure 2 shows the overall percentage of volunteers who demonstrated cognitive decline at each time point compared to those who did not show a significant change in their cognitive function from baseline in the tests as per the ISPOCD trial definition of cognitive decline.

**Table 1:** Repeated measures analysis of change in accuracy from volunteer’s baseline for each time point.

Time point	Event	Accuracy, mean change from baseline (95% CI)	*P* value
2	30 minutes after Trendelenburg positioning	−0.04 (−0.072 to 0.001)	.06
3	60 minutes after Trendelenburg positioning	-0.01 (−0.05 to 0.03)	.66
4	90 minutes after Trendelenburg positioning	0.01 (−0.03 to 0.05)	.67
5	120 minutes after Trendelenburg positioning	0.02 (−0.02 to 0.05)	.44
6	150 minutes after Trendelenburg positioning	0.002 (−0.35 to 0.04)	.91
7	Supine (after 180 minutes in the Trendelenburg position)	0.02 (−0.01 to 0.06)	.20
8	30 minutes after supine positioning	0.01 (−0.03 to 0.04)	.82
9	Sitting up (60 minutes after supine positioning)	0.02 (−0.02, 0.06)	.29

**Table 2:** Repeated measures analysis of the change in response time from baseline for each time point.

Time point	Event	Accuracy, mean change from baseline (95% CI)	*P* value
2	30 minutes after Trendelenburg positioning	−0.04 (−0.11 to 0.04)	.34
3	60 minutes after Trendelenburg positioning	−0.08 (−0.16 to 0.01)	.03
4	90 minutes after Trendelenburg positioning	−0.11 (−0.18 to −0.03)	.004
5	120 minutes after Trendelenburg positioning	−0.15 (−0.22 to −0.07)	<.001
6	150 minutes after Trendelenburg positioning	−0.14 (−0.22 to −0.07)	<.001
7	Supine (after 180 minutes in the Trendelenburg position)	−0.19 (−0.26 to −0.11)	<.001
8	30 minutes after supine positioning	−0.15 (−0.22 to −0.7)	<.001
9	Sitting up (60 minutes after supine positioning)	−0.16 (−0.23 to −0.08)	<.001

**Table 3:** Average response time to tests at each time point with the percentage of volunteers with cognitive decline from their own baseline (N=15).

Time point	Event	Volunteers with normal cognitive function, n (%)	Volunteers with cognitive decline, n (%)
2	30 minutes after Trendelenburg positioning	12 (80)	3 (20)
3	60 minutes after Trendelenburg positioning	11 (73)	4 (27)
4	90 minutes after Trendelenburg positioning	10 (67)	5 (33)
5	120 minutes after Trendelenburg positioning	11 (73)	4 (27)
6	150 minutes after Trendelenburg positioning	11 (73)	4 (27)
7	Supine (after 180 minutes in the Trendelenburg position)	9 (60)	6 (40)
8	30 minutes after supine positioning	10 (67)	5 (33)
9	Sitting up (60 minutes after supine positioning)	9 (60)	6 (40)

## Discussion

### Principal Findings

Our study showed a decline in the cognitive function of participants following Trendelenburg positioning. Each volunteer’s cognitive function was assessed at various time points against his or her own baseline cognitive performance. The overall percentage of volunteers with cognitive decline increased to 40% (6/15) after 3 hours in the Trendelenburg position. After 1.5 hours, 33% (5/15) had cognitive decline, but this reduced to 27% (4/15) after 2 hours. This could be due to adaptation to being placed in the Trendelenburg position as per the Monroe-Kellie doctrine or possibly due to 5 of the 15 volunteers requiring a toilet break between 90 minutes and 2 hours into the test [17].

Once the participant sat up (after 3 hours in the Trendelenburg position and 1 hour in supine position), the initial tests revealed another slight increase in the number of participants with overall cognitive decline from 33% (5/15) to 40% (6/15). Many physiological changes initially occur in an individual when sitting from a supine position. Deegan et al recruited 19 healthy volunteers and induced transient hypotension while in both the seated and supine positions and measured mean arterial pressure and cerebral blood flow in the middle and anterior cerebral arteries along with cerebral autoregulation response. They found that autoregulatory responses were worse in the seated position in both the anterior and middle cerebral arteries, which was thought to be due to the hydrostatic gradient that occurs when in a seated position [18]. Previous studies have shown that cerebral autoregulation is dependent on vascular tone [19]. Deegan et al found that a drop in cerebral perfusion pressure leads to dilatation of the cerebral vessels, which results in reduced cerebral vascular resistance. They also looked at the theory of reduced cerebral perfusion pressure resulting from a shift in the autoregulatory curve to the right. However, the subjects included in their study did respond with an increased heart rate, suggesting a sympathetic response that should result in vasoconstriction and, therefore, an increase in cerebral vascular resistance [18]. This could explain the increase in the percentage of volunteers with cognitive decline that occurred at time point 8.

A break was taken by some volunteers between time points 3 and 4, which lasted for a maximum of 5 minutes. The volunteers then returned to the Trendelenburg position. This break could explain the reduction in the percentage of volunteers with cognitive decline at time point 4. The resulting reduction in cognitive decline that may have occurred following a short period in the upright position was comparable to the worsened cognitive decline that occurred when in the sitting position, which further supports the reduced cerebral perfusion pressure that has been shown to occur when in the seated position versus the standing position. Patients undergoing left-sided laparoscopic resections are often placed in either a modified lithotomy position (with the legs slightly flexed) or the Lloyd-Davis position. Further studies to reassess the response of a standing or head-up tilt position on recovery of cognitive function would be of clinical benefit.

There were limitations to this study, which included a bathroom break that may have affected the results achieved at time point 4. Test fatigue could also be a contributing factor, with repetitive tests being conducted in such a short time period. A repeat set of tests 24 hours after the end of the study would possibly have been beneficial for assessing the clinical impact.

A further limitation is the lack of a clear definition for POCD. ISPOCD is the largest study in this area so far, but it treats POCD as a binary definition. The clinical implications and impact on daily life of these definitions need to be further evaluated and defined along with standard tests that should be used. The high percentage of cognitive decline could be due to the sensitivity and number of the cognitive tests that were used in our study.

### Conclusion

The results of our study indicate that Trendelenburg positioning leads to cognitive decline. A finding of potential clinical significance was that when the volunteers sat up from supine position, there was an increase in the number of volunteers with cognitive decline. This suggests that in a clinical setting, simply reducing the tilt of the table when the patient is in the modified lithotomy or Lloyd-Davis position may not be beneficial as this would most likely mimic the physiology of sitting and could further impair cognitive function due to reduced cerebral vascular resistance [18]. Further studies to assess the effect of a “break from the Trendelenburg position” while in the modified lithotomy or Lloyd-Davis position versus supine position would be clinically relevant.

## Abbreviations

ISPOCD: International Study of Postoperative Cognitive Dysfunction
LDT: lexical decision-making task
POCD: postoperative cognitive decline
RT: response time
